# Integrating remote datasets to identify precontact architecture and settlement patterns along the Wampu River system, Eastern Honduras

**DOI:** 10.1371/journal.pone.0335239

**Published:** 2025-11-12

**Authors:** Anna S. Cohen, Juan Carlos Fernandez-Diaz, Elizabeth Groat, Quinn Eury

**Affiliations:** 1 Department of Anthropology, Florida State University, Tallahassee, Florida, United States of America; 2 National Center for Airborne Laser Mapping, University of Houston, Houston, Texas, United States of America; 3 Stell Environmental Enterprises, Inc., Mountlake Terrace, Washington, United States of America; 4 Anthropology Program, Utah State University, Logan, Utah, United States of America; University of Michigan, UNITED STATES OF AMERICA

## Abstract

In our era of remote sensing archaeology and legacy datasets, multiple lines of evidence should be integrated to establish a large-scale view of threatened Indigenous landscapes. This study brings together several datasets derived from airborne lidar, satellite imagery, and pedestrian survey to present the first large-scale (>650 km^2^) synthesis of the settlement archaeology of the Wampu River system, a part of eastern Honduras with limited archaeological work. The purpose of this study is to: 1) connect pedestrian and airborne lidar surveys within the Wampu River system; 2) identify patterns in archaeological sites and architecture; 3) characterize the anthropogenic landscape of the region, and to suggest areas for future work in this understudied part of the Americas. Results include the identification of 72 archaeological sites, 55 of which were previously unreported, and the creation of a basic classificatory scheme for site organization. Other findings include the preliminary observations that water, topography, and seasonality likely structured settlement patterns, and that there are emerging patterns in site orientation. We also consider how eastern Honduras could contribute to broader discussions about tropical settlement patterns. Overall, this study demonstrates that eastern Honduras has the potential for future research identifying extensive settlements in the centuries before European arrival, thus contributing to a more complex understanding of the extent and diversity of Indigenous populations in the Americas.

## Introduction

The uses of remote sensing data derived originally from airborne and satellite imagery, later from airborne lidar, satellite radar, and currently with miniaturized passive and active sensors from remotely piloted or autonomous drones, have changed the way that archaeologists collect and interpret landscape-level data for a region [1–5], but high resolution wide-area (>100 km^2^) data collection remains costly [6]. Smaller remote datasets can be more accessible, however, and there are numerous smaller datasets that can be integrated to improve our understanding of anthropogenic landscapes and archaeological settlement patterns. When combined with information from previous fieldwork, these datasets help to capture threatened landscapes especially in parts of the world that experience ecological and political instability.

**Fig 1:**
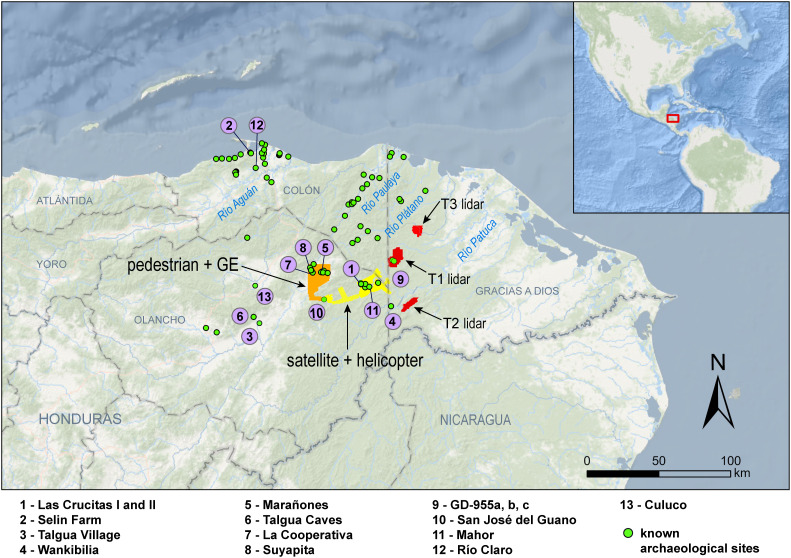
Overview map of eastern Honduras. Included here are the locations of the datasets, sites mentioned in the text, and other known archaeological sites that were georeferenced. GE = Google Earth. Basemap data from OpenStreetMap.

This study integrates several remote sensing datasets, including airborne lidar and satellite images, to present the first large-scale (>650 km^2^) synthesis of the settlement archaeology of the Wampu River system, a part of eastern Honduras with a long but uneven history of research (Fig 1). Eastern Honduras, also referred to in the literature as northeastern Honduras [7], is itself a relatively under-researched part of the Americas that was part of a broader interaction sphere in precontact Central America in which populations shared linguistic, ethnic, and genetic heritage [8,9]. The existing work in the region suggests that precontact Indigenous groups created monumental architecture and material culture, but they also selectively and strategically borrowed from cultural traditions to the north and south [10]. These unique cultural processes mean that archaeological data from eastern Honduras will enrich discussions about tropical settlement patterns and the ongoing reevaluation of precontact landscapes all over the Americas [5,11–13].

The purpose of this study is to:

1) use high resolution (<1 m) spaceborne optical imagery to connect two previously published settlement surveys on opposite ends of the Wampu River system – a pedestrian survey of a portion of the central Culmí Valley [14] and a 2012 airborne lidar survey in the Mosquitia region [15] – to provide a comprehensive, but preliminary, archaeological survey of this broad region;
2) identify patterns in the location and layout of monumental archaeological sites and settlement patterns within the Wampu River system;
3) compare and contrast patterns in the location and layout of monumental architecture within the Wampu River system, and to other adjacent cultural regions;
4) characterize the broader anthropogenic landscape and suggest avenues for future work in this understudied part of the Americas.

By incorporating data from pedestrian survey and testing, airborne lidar, satellite imagery, and helicopter surveys, combining previous research with newer remote sensing data collections, we synthesize >650 km^2^ of archaeological surveys in eastern Honduras. In doing so, we show how smaller remote and disparate datasets can be useful for identifying and assessing anthropogenic landscapes in regions that may be challenging for sustained large-scale fieldwork. With our analysis of geospatial data, we remotely identified a total of 72 archaeological sites in the Wampu River region, 55 of which we were not able to find a correspondence for in the archaeological literature. We highlight patterns in the site configurations, including discussions of site organization and morphology, location, and orientation. Methodologically, we show how the use of legacy and more recent pedestrian and remote survey data can be combined to identify settlement and architectural patterns in regions and at sites specifically. This is critical in our era of large-scale data collection, along with the extensive legacy data available in grey and published literature, and in museum collections [16–18]. Such strategies are particularly valuable in regions like eastern Honduras, where dynamic environmental and socio-political processes can pose challenges to heritage preservation, and where limited archaeological survey data may nonetheless hold key insights for understanding landscape patterns at a continental scale. Conceptually, this study adds to ongoing discussions about the extent of precontact settlements in the Americas. We show that research in eastern Honduras has the potential to identify extensive settlements in the centuries before the arrival of Europeans, thus contributing to a more complex understanding of the extent and diversity of Indigenous populations in the Americas.

### Geographical and cultural context

The location of this study is in eastern Honduras, defined here as parts or all of the departments of Atlántida, Colón, Gracias a Dios, Olancho, and Yoro (Fig 1). Here the focus is on the Wampu River system, a major water body in eastern Honduras that has been partially investigated, both formally and informally, and that shows evidence of extensive archaeological occupation (Fig 1). The Wampu River system was selected because of its archaeological potential, previously published survey datasets, deforestation that allows for the identification of archaeological sites in low-cost, very- high-resolution (VHR) satellite imagery, and because it demonstrates how integrating disparate datasets can provide important information about a region. The river flows southwest along the southern and eastern edges of the Culmí Valley, in the eastern part of the department of Olancho, and it drains into the Patuca River. Numerous creeks and small rivers crosscut the valley floor, creating rolling and steep hills and long flat ridges in between; elevations range from 400–600 m asl on the valley floor, and up to 1500 m in the mountains [14,19]. Geologically, the valley is on the edge of highlands formed of metamorphic rock like slate, mica, schists, and quartzite [20]. It forms the western boundary of the Mosquitia, the coastal lowland plain and inland uplands covered in tropical forest and swampy savannas in the departments of Colón, Gracias a Dios, and Olancho. Soils in eastern Honduras are usually acidic, though richer alluvial types form terraces along waterways like the Wampu [21].

Indigenous communities living in or near the Wampu River region today and those in the past are related to Circum-Caribbean cultures. The Pech and Tawakha Indigenous groups have been living in eastern Honduras since at least late precontact periods, along with more recent Miskito communities [22–25]. Pech communities speak Chibchan languages, which are thought to have emerged from what is now Costa Rica and Panamá, while Tawakha is an ethno-linguistic subgroup of Mayangna peoples, who today live primarily in Nicaragua. As discussed below, the ethnohistoric record from the early contact period includes numerous references to Pech, Tawakha, and other Indigenous communities throughout eastern Honduras that engaged in dynamic cultural practices that were different from those observed in adjacent regions [19,24]. Based on ethnohistoric references and various iconography and monumental architecture, it has been suggested that certain larger sites in eastern Honduras were influenced by migrations of Nahuatl speakers, possibly from central Mesoamerica, around the time of European contact [26]. Early European accounts mentioned Nahua enclaves along the northern coast of Honduras and to the west, and Nahuatl toponyms were recorded by Cortés in the lower Aguán Valley [27], but this idea has not been tested archaeologically. Today, the landscapes of the Wampu River system and much of eastern Honduras are increasingly threatened due to intersecting human and environmental pressures – including political instability, deforestation, and weak regulatory enforcement – factors that are often exacerbated by the legacy and ongoing impacts of foreign geopolitical interests in the region [28].

### Archaeological context and previous research

In this study, we integrate rough site location and/or layout information from previous research in the region with more recent remote sensing data collections. Early archaeological work in eastern Honduras mainly consisted of excursions in the 1920s and 1930s led by U.S.-based individuals or museums [25,29–32]. In the 1970s, Paul Healy’s [33–35] projects surveyed and excavated sites in eastern Honduras and built upon Jeremiah Epstein’s [36] ceramic research to develop a chronology that is still used today (Table 1). Reviews of this early work are available in [7,35].

**Table 1:** The chronology and available radiocarbon determinations in published literature for eastern Honduras. Note that the majority of the dates that have helped to develop this chronology (along with pottery and architecture studies) are from Selin Farm and other sites north of the Wampu River system. Only one site in the Wampu River system has a published date that we know of, La Cooperativa [14]. At the time of submission, these were the dates that were available to us. Since the manuscript was accepted, we have become aware of additional radiocarbon dates: one set has been reported in oral presentations by IHAH (site GD-955, Proyecto Kaha Kamasa/Ciudad Blanca) and another set of 35 dates is reported in F. Fecher ’s dissertation on the Caribbean coastal site of Guadalupe.

Dates	Mesoamerican cultural period	Chronological period^a^	Eastern Honduran cultural period^b^*	Social-political developments	No. ^14^C Dates	Site(s)	References
AD 1250–1521	Late Postclassic	Period 6b	Late Cocal	Larger sites and monumental architecture	2	La Cooperativa Rio Claro	[14,34]
		Period 6a	Early Cocal		2	Rio Claro	[34]
AD 900–1250	Early – Middle Postclassic	Period 6a Period 5	Early Cocal Transitional Selin	Regional prosperity Increasing populations, architecture	6	Selin Farm El Cafetal Rio Claro	[14,34,48]
AD 700–900	Terminal Classic	Period 5	Basic-Transitional Selin		8	Selin Farm Talgua Village	[34,41,48,50]
AD 250–700	Classic	Period 5	Basic Selin	Complex site planning Increasing populations	22	Selin Farm Altas de Subirana El Garrapatero Buena Vista El Cafetal C. de Río de Talgua	[14,34,41,48,50]
		Period 4b	Early-Basic Selin		3	Selin Farm	[48]
		Period 4b	Early Selin		10	Selin Farm	[34,48,50]
400 BC – AD 250	Late Formative	Period 4b	Cuyamel	Ritual pilgrimages Long-distance trade	2	Selin Farm	[48]
1200–400 BC	Middle Formative	Period 3-4a	Cuyamel	Ritual pilgrimages Cave ossuaries	4	C. de Río Talgua C. de Río Talgua (Arañas)	[41,45]
2500–1400 BC	Early Formative	Period 3	--	Ritual pilgrimages Cave ossuaries	1	C. de Río Talgua	[41]
				**TOTAL DATES: 60**		**TOTAL SITES: 10**	

^a^This is based on Healy’s [35] Honduran chronology that draws from the broader Central American (referred to as the Intermediate Area by Healy) chronology.

^b^This chronology was initially developed by Epstein [36], but was later expanded upon by Healy [35] and ceramic analyses [51].

More recent research has incorporated updated survey methods. In the 1980s, following encouraging reports of site identifications by Operations Drake and Raleigh, the Honduran Institute of Anthropology and History (IHAH) documented two large sites at las Crucitas, near the confluence of the Wampu and Aner Rivers [37]. Christopher Begley’s Proyecto Rio Plátano was initiated in 1991 and documented around 125 sites in the central Culmí Valley (the western stretch of the Wampu River and the headwaters of the Plátano and Paulaya Rivers) with the help of Pech collaborators [14]. Other work in the Mosquitia region includes airborne lidar scans in 2012 and survey and test excavations in 2015 and 2016 [15,38]. Finally, there are numerous other notable sites throughout the broader eastern Honduras region mentioned in academic and associated grey literature, demonstrating the potential for widespread archaeological settlements throughout the precontact periods [10,16,39–49].

The Wampu River system chronology is mainly based on relative and absolute dates from outside the region, including from Healy [35], Begley [14], and other studies [7,36,41,48,50,51] (Table 1). The chronology includes 60 radiocarbon determinations from 10 sites, in addition to ceramic and other artifact associations. The earliest dated architecture is from the Selin period (AD 300–1000), best characterized by a series of loosely organized mound groups at the sites of Selin Farm and Talgua Village, both of which are outside of the Wampu region. In the subsequent Cocal period, AD 1000–1530, larger sites and monumental architecture have been documented, including plazas, ballcourts, and raised causeways such as at las Crucitas, Wankibila, and Marañones [44]. Healy [35,52] has argued that these kinds of sites reflect social hierarchies and may represent chiefdoms, or ranked societies with a headperson and evidence of tribute or trade, as observed by early Europeans in the region. These processes of increasing settlement size and construction contrast with the depopulation of large settlements in western Honduras around the late Classic period, along the southeastern Maya periphery [10,35,53,54]. Although not in the Wampu River region, research at the Formative period Talgua Caves [41,45] demonstrates that there was long-term use of landscapes and caves in eastern Honduras. These findings demonstrate the great need for further research to document the extent and variation of anthropogenic features on the landscape.

## Materials and methods

Due to the remoteness and extent of the Wampu River region, this study incorporates remote sensing datasets obtained from different sensors and platforms. However, these remote datasets are contextualized through data from Begley’s [14] pedestrian survey of 100 km^2^ in the central Culmí Valley, with the goal of aggregating the datasets and identifying any patterns in precontact architecture and settlement patterns in the Wampu River region (Table 2). This work was based on the visual inspection of publicly accessible remote sensing data, mainly VHR satellite imagery; thus, no permits were required, and the findings as well as copies of relevant materials are shared with the Instituto Hondureño de Antropología e Historia (IHAH). When visually inspecting the remote sensing imagery, a confidence level was assigned to each feature and site that could be archaeological. Since these are mostly remotely-identified sites, a site was defined as either one clear mound or multiple mounds (or features); however, as further discussed below, we cannot assign function to the sites at this point. Sites with high level confidence are ones that have multiple mounds arranged in geometric patterns, or that were identified on several different images or datasets. Sites were noted as medium confidence if features could not be verified on multiple images or if the features appear blurry or faint. Additionally, medium confidence sites could be sites where only a single mound was clearly visible and/or one that exhibits considerable volume. Finally, a site was considered low confidence if features (single or multiple mounds) could not be verified on multiple images, if they were blurry or faint, and/or if the features were not in a geometric arrangement. In S2 File, KMZ files that can be opened on Google Earth include examples of features that we identified at high and medium confidence levels, as well as features that have been identified at low confidence level.

**Table 2:** Datasets used in this study.

Dataset	Strengths	Limitations
Historical archaeological maps/ descriptions	Best source of historical record	Limited details Poor geolocation accuracy
Topographic maps 1:50,000	Excellent geolocation accuracy	Limited number of sites marked General outline for site, but no architectural information
Published site layouts (e.g., Río Claro, Wankibila, Crucitas de Aner I & II, Talgua Village, Chichicaste)	Available for large key sites in the region	No geolocation Subjective interpretation of sites Little to no topographic information
Pedestrian survey [14]	Systematic pedestrian survey, shovel tests, limited excavation	Mostly limited to the central portion of the Culmí valley
Airborne lidar survey (UTL 2012)	147 km^2^ of high density lidar (> 15 pulses/m2) coverage of 3 valley systems, plus 2 other spot acquisitions Complete representation of landscape and vegetation structure	Moderate to low fidelity given dense forest cover Most smaller structures of a site are not identifiable Field validation of a small section
Oblique helicopter photos (2015/2016)	Very high detailed view of key sites without vegetation	Limited number of sites along the flight path
Commercially available satellite data MS/SAR (very-high-resolution imagery < 1 m)	Moderate geolocation accuracy Possible to identify large sites in areas without vegetation Relatively inexpensive	Most smaller structures of a site are not identifiable Analysis requires prior experience for identification of sites
Open imagery sources/ Google Earth/ Bing/ etc	Free Good historical record (multiple images spanning years to decades for one area)	High resolution images (<1 m) are only available in areas with dense human habitation Limited ability to manipulate the imagery on browser or standalone client

**Fig 2:**
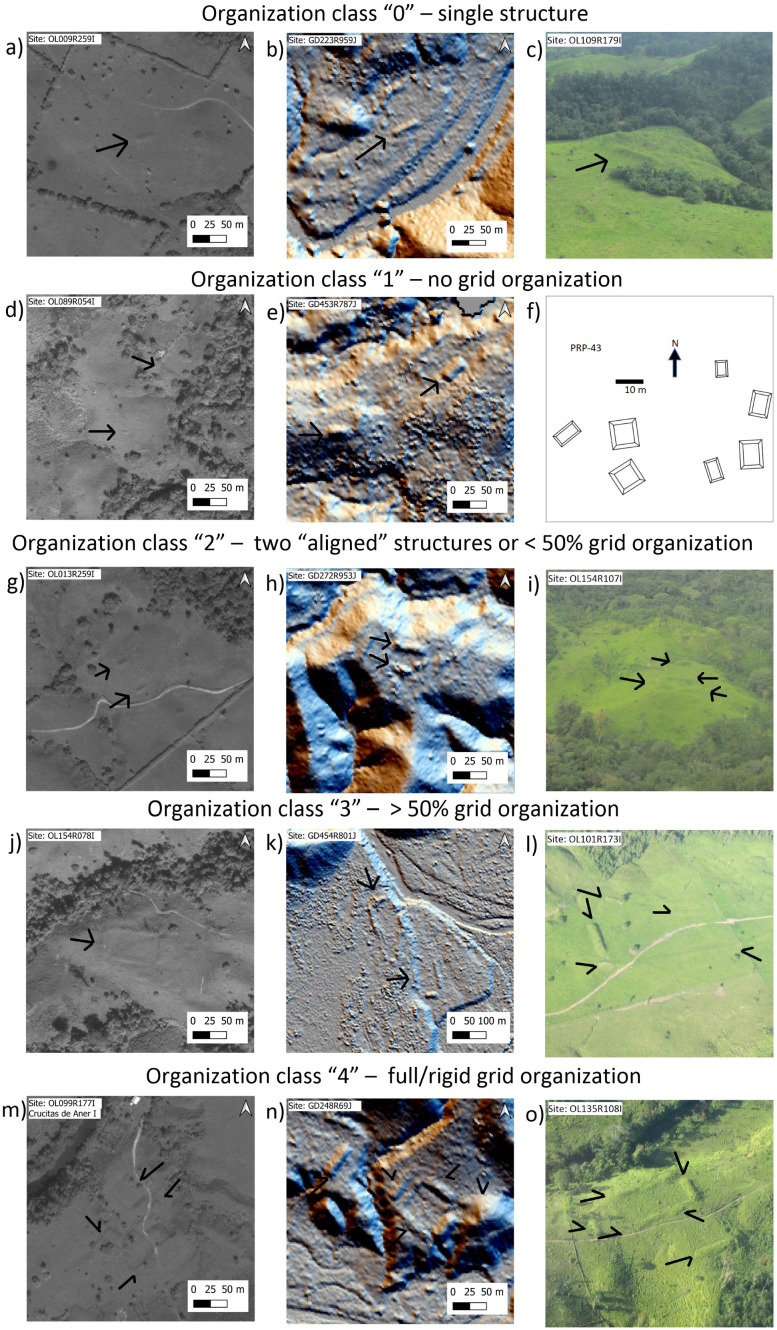
Organizational classes 0-4 used in this study. Images a, d, g, j, and m are from WorldView 2 and 3 satellite multispectral data (imagery used with permission from Maxar Technologies ©2025); b, e, h, k, n are from airborne lidar scanning (image produced by authors from lidar data courtesy of UTL productions); c, i, l, and o are helicopter photos (taken by the authors); and image f is redrawn from [14].

**Fig 3:**
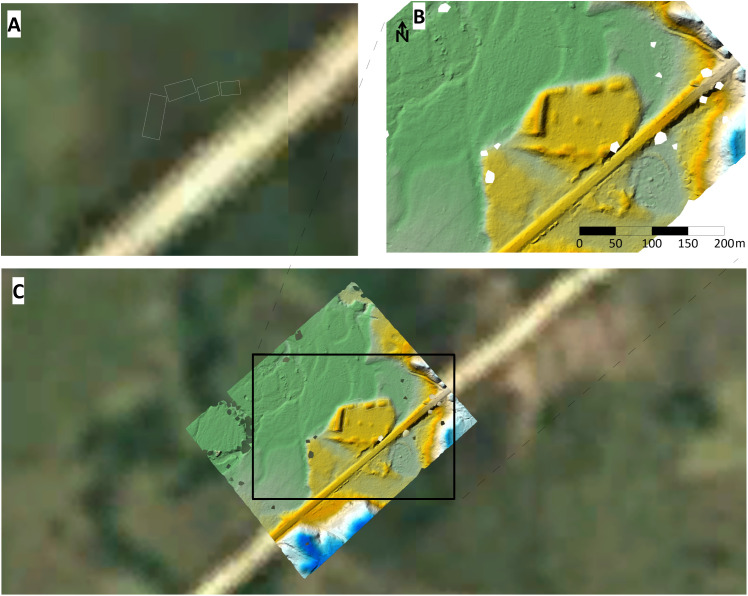
Comparison of remote datasets for identifying the archaeological site of Culuco (No. 13 in Fig 1). A) Landsat image from 1985, B) UAV lidar scan; C) Lidar scan over Landsat image. See also S1 File.

At all sites, architectural layouts were assigned to basic organizational classes, 0–4 (Fig 2), based on whether the architecture appeared as though it were constructed on a grid system. Here we use the term grid to refer to whether the architecture shows orthogonal or ortho-parallel characteristics. If architecture exhibited right angles, parallelism, and/or the arrangement of structures in a visibly organized manner, then this could indicate intentionality on the part of the builders. The focus on the intentionality of architecture as an analytical tool allows us to ask whether individual agents or communities of builders standardized construction, worked in communities of practice, and/or focused on certain feature types [55–57]. Notably, this classification system avoids functional types, including common architectural features in Mesoamerica, such as plazas, ballcourts, and altars. Assigning function and a more formal architectural typology are future steps; however, this will require higher fidelity geospatial data, such as lidar, which can provide more complete representations of the site architecture (for a comparison, see Fig 3), in addition to systematic field investigations. For now, as the architecture is assigned to general organizational classes, sites can be compared within the region and to other regions with large datasets such as the Maya cultural area, thus setting up a baseline for future quantitative work.

Sites were assigned to five different classes, 0, 1, 2, 3, or 4. The 0 class was used to indicate single mounds. Sites with more than one mound, little or no organization, and no clear grid system, were classified as 1. Sites consisting of two similarly or differently sized mounds running in different directions fell into this category. Sites with some organization, though not on a grid system, were classified as 2. For a site to fall into this category, fewer than 50% of the architectural features must show organization in some way. Sites with two mounds of similar sizes with axes in some sort of arrangement (parallel, co-axial, perpendicular) were also classified as 2. Sites with at least 50% of the features organized on a grid system with right angles or parallel structures were classified as 3; this class also includes sites where the mounds are not perfectly rectilinear. If there were multiple axes, such as three parallel mounds in a row, these were also classified as 3. Finally, sites that were fully constructed on a grid, including right angles and long parallel mounds, were classified as 4. Class 4 sites represent the most complex arrangements in the Wampu River region. Once sites were assigned to these classes, they were examined with descriptive statistics to identify any patterns within the region.

The locations of these sites in relation to water bodies was explored to identify and evaluate any patterns in the placements of sites and to compare to existing research in Mesoamerica and elsewhere. Anecdotally, it has been observed that sites in eastern Honduras are located near rivers or streams, usually on the second or higher terrace above the flood plain, and also close to the intersections of rivers and streams [58]. The lidar and satellite imagery datasets, together with GIS software, allowed us to quantify and analyze each site’s proximity to rivers or streams. To do this, we measured two distances: a) the shortest distance from the approximate centroid of the site to the centerline of the closest river or stream irrespective of its order, and b) the distance to the closest river of medium or high order. In hydrology, stream order is a way to categorize the hierarchy of a stream within the watershed [59]: first-order streams have low water flow and no tributaries, second-order streams are formed when two first-order streams meet, and so on. We did not have access to a GIS layer showing the numerical order for the rivers and streams in our area of study, so we manually assigned each stream a low, medium, or high order category based on the width of the stream on the imagery and our rough assessment of the hydrological basins based on the 1:50,000 topographic maps. The Wampu River is the highest order stream in our research area, while its named tributaries such as the Guano, Lagarto, Aner, Pao, and Ahuas Rivers were considered medium order streams. Streams that connect to these medium order rivers were designated as lower order. Finally, zero order areas were identified in the satellite images as areas where the watershed starts to form.

As another way of comparing this dataset to better-known features in southeastern Mesoamerica, we examined architectural orientations for any patterns, potentially pertaining to seasonality and ritual. As a starting point, we applied similar analyses to those that have been used in the southern Gulf Coast of Mesoamerica [60,61], despite being limited to a lower resolution, fidelity, and scale of lidar data. We recorded three kinds of azimuth measurements for the remotely identified sites: a) azimuth for the main (longest) axis for the site, b) azimuth for the largest structure at the site, and c) azimuth for the main site axis, taking as the origin the largest structure of the site. In order to have comparable and aggregable measurements, we recorded the above-described azimuths in the ranges of 270° (West of North) to 360° (North) to 90° (East of North).

The combined datasets in this study include information from early archaeological work in the Wampu region [31], but also from Begley’s [14] more recent pedestrian survey, airborne lidar collections [15], and VHR, commercially available (publicly-accessible) satellite imagery. Here we summarize each dataset (see Table 2, which includes the strengths and limitations of each dataset). Also used were historical maps and descriptions, topographic maps, and helicopter photos to help fill in gaps about existing sites and architectural categorizations that are referenced where appropriate.

### Pedestrian survey

**Fig 4:**
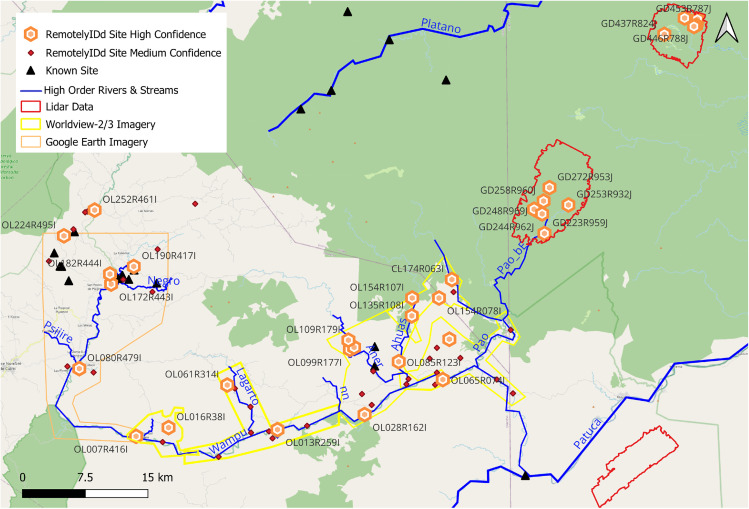
Sites remotely identified in the Wampu River region, including those previously identified via pedestrian survey and recent lidar scans and field verification. Basemap data from OpenStreetMap.

In the 1990s, Begley [14] documented over 125 archaeological sites in the Culmí Valley with the help of the Pech and other local informants (Figs 1 and 4). The Rio Plátano Project was begun as part of an initiative by IHAH to encourage research in the region. The first phase consisted of a key site survey (defined as “obtaining information about and visiting known sites with local residents” (p. 42)) and subsurface testing program aimed at identifying non-mounded sites, while the second phase consisted of a key site survey further east in the Mosquitia. The overall pedestrian survey of 100 km^2^ included roughly 87 sites with monumental architecture, such as La Cooperativa, Marañones, and Suyapita, the largest known sites in the Culmí Valley, as well as other smaller sites with visible architecture along tributaries of the western Wampu and the headwaters of the Plátano River. Sites consisted of visible earthen mound and/or stone architecture, ceramic scatters, or both [14]. Begley provides line drawings for each monumental site with architecture, and these were compared with architecture visible in remote sensing data for the site organization classification (Fig 2). Begley’s line drawings were digitized in a CAD software, with measurements recorded for each mound, and orientation recorded for the main site axis. Some of Begley’s site data included radiocarbon dates from test excavations, or discussion of occupation ranges from architecture and ceramic comparisons in the region [14]. While these pedestrian survey data lack the embedded geolocation information present in modern remote sensing data, Begley’s information serves as a foundation for situating and interpreting recent data collection into a robust framework that integrates information about site layouts, number of structures, surface extent, and site orientation. Further, in contrast to the satellite imagery, which only provides useful settlement and site information in deforested areas and thus just a sample of the archaeological landscape, the main pedestrian survey provides a complete and continuous map of the material culture within the research area. As a control dataset, the pedestrian survey can be used to extrapolate and contextualize the information obtained from the non-continuous satellite survey.

### 2012-2016 Mosquitia projects

Between 2012 and 2016, as part of two related projects in the Mosquitia region, 147 km^2^ of airborne lidar were collected in three main survey areas called T1, T2, and T3 (Figs 1 and 4), and a few other opportunistic targets (T4 and Cerro Pomokir). These areas were selected because there was little or no previous published research in the area. Over a two-week period, seven flights were carried out for 32.1 hours and 8.4 hours of Laser-On-Time, for a total of 3.5 billion laser pulses. Only 2.9 billion pulses were processed for 4.5 billion returns; of these, 87 million (1.9%) were classified as ground returns. Bare-earth digital elevation models (DSMs) were created from these filtered ground returns at 1-meter cell spacing using procedures described in [62], in addition to shaded relief and other products (additional details are in [15]). Using contour maps and shaded relief (single and multi-illumination angles) products derived from the scans, in 2015 and 2016, remote and field mapping of the T1 region identified 19 sites, with one large site (GD-955 or GD248R969J) consisting of monumental architecture, water management systems, terraces, and artifacts, including a cache of stone carved “metates” and ceramics [15]. Field verification and excavation in 2015 and 2016 at site GD-955 resulted in nearly 200 complete or fragmented stone metates with zoomorphic and anthropomorphic iconography. Initial observations about this site suggest dispersed communities and hints of *milpas* or kitchen gardens adjacent to dwellings, and the exploitation of larger catchment areas to diversify diet and other resources.

The T1 and T3 datasets in this study are in river systems that drain into the eastern Wampu River system through the Pao River. The lidar-derived products including terrain hillshades were used to reexamine previously identified anthropogenic features. Even if a feature and site were identified previously [15], they were reevaluated and, in some cases, reclassified. If something was unclear, a profile of the point cloud was evaluated in detail to determine if it was human-modified or natural geomorphology.

### Very-high-resolution (VHR) commercial multispectral satellite imagery

In recent years, satellite imagery has become increasingly useful in tropical environments due in part to the rapid rate of deforestation [63–65]. Here, 297 km^2^ of WorldView 2 and 3 satellite panchromatic and multispectral data from the Wampu River system and adjacent regions were purchased, both to assess their usefulness for site identification based on known site locations, and to see if additional features or sites could be identified. Panchromatic images at 50 cm resolution were purchased for five different areas (see S2 File for outlines of these regions), targeting deforested areas and mostly cloud-free images. Data from these areas represent a continuous section of the Wampu River and its tributaries (the Pao, Aner, Ahuas, and Lagarto Rivers). A 500 m x 500 m grid was created to cover the imagery footprint, which was then used to guide a cell-by-cell visual inspection of the images. Inspection was conducted independently by three individuals (co-authors), recording any potential features of interest in a GIS shapefile. Upon completion, the features were collectively discussed and classified as likely of anthropogenic or natural origin.

### Freely accessible Google Earth high-resolution historical imagery

For some areas in eastern Honduras that include modern, medium-sized rural settlements (*pueblos* and *caserios*), high-resolution (~0.5 m) pan-sharpened RGB natural color imagery is available through the Google Earth imagery archive. This is the case for the section of the Wampu River that runs through the Culmí Valley. We visually inspected 264 km^2^ of Google Earth Imagery (including different images for multiple dates) from the northern boundary of Begley’s [14] pedestrian survey block south to the Wampu canyon, connecting to the commercial imagery around the village of San José del Guano (see Figs 1 and 4 and S1 File).

### Opportunistic helicopter surveys

During the ground validation and excavation of the Mosquitia lidar scans in 2015 and 2016, the field crew was ferried by helicopter from the city of Catacamas to site GD-955/GD248R969J. Similar to satellite imagery, helicopter photography is an increasingly useful dataset in tropical regions, as deforestation exposes more and more monumental sites. Oblique photos taken from the helicopter were geolocated and used as an additional source of data for the identification of sites, and for comparison and validation of satellite imagery. These airborne photographs are higher resolution and usually show more details and features of sites than satellite images. The available spatial coverage is, however, limited to a narrow strip around the flight path, so the results are most useful when combined with other remote datasets.

## Results

The purpose of this study is to aggregate and synthesize remote and pedestrian datasets over 650 km^2^ along a partially explored river system, and to identify any new sites and patterns in eastern Honduran architecture. Our results include the identification of previously unrecorded archaeological sites, some common architecture and site types, general observations about site locations on the landscape, and patterns in site orientation. Here we present the results in terms of site identification, architectural and site organization, site distance to water sources, and site orientation.

### Identification of archaeological sites

The combined datasets show a total of 72 archaeological sites (34 at high confidence and 38 at medium confidence) in the Wampu River region, 55 of which are not present in the existing archaeological literature (Fig 4). The confidence level criteria were described in the Materials and Methods section. For traceability and reference, each remotely identified site was assigned a unique ten-character identification (ID) code. The first two characters in the code represent the largest Honduran territorial division (department) and the eight remaining alphanumeric characters are coded representations of the estimated centroid geographic coordinates. Table 3 summarizes the sites by sub-region (e.g., central or north Culmí Valley), imagery type, and confidence level. The main sub-regions considered here include:

**Table 3:** Breakdown of archaeological sites remotely identified by human visual inspection by sub-region, type of remote sensing imagery used, and documentation status (previously reported or uncertain).

Inspection Subregion^a^	CCul	NCul	SCul	CWam	GD T1	GD T3	CL T4	Total
**Area km** ^**2**^	105	6.4^d^	159	297.6^e^	62.7	26.6	4.3	661.6
**Primary data** ^**b**^	GEI	GEI	GEI	WV	aLidar	aLidar	aLidar	
**Secondary data**	CBD/ TM	TM	TM	TM/ HP	–	–	WV	
**High confidence** ^**c**^	UC: - PR: 4	UC: 1 PR: -	UC: 1 PR: -	UC: 11 PR: 3	UC: - PR: 7	UC: 6 PR: -	UC: 1 PR: -	UC: 20 PR: 14 TT: 34
**Medium confidence**	UC: - PR 3	UC: 4 PR: -	UC: 3 PR: -	UC: 28 PR: -	UC: - PR: -	UC: - PR: -	UC: - PR: -	UC: 35 PR: 3 TT: 38
**Total**	UC: - PR: 7 TT: 7	UC: 5 PR: - TT: 5	UC: 4 PR: - TT: 4	UC: 39 PR: 3 TT: 42	UC: - PR: 7 TT: 7	UC: 6 PR: - TT: 6	UC: 1 PR: - TT: 1	**UC: 55** **PR: 17** **TT: 72**
**Previously reported, but not identified**	79 including one in topo map			4				

^a^Subregion: CCul = Central Culmi (Begley pedestrian survey), NCu = North Culmi (North of Begley pedestrian survey), SCul = South Culmi (South of Begley pedestrian survey), CWam = Central Wampu (East of el Guano to intersection with Pao River), GD T1 = Gracias a Dios T1, GD T3 = Gracias a Dios T3, CL = Colon T4.

^b^Data sources: GEI = Google Earth historic pansharpened RGB imagery, WV = Worldview –2/3 panchromatic, aLidar = airborne lidar, CBD = Begley Dissertation, TM = Topographic Maps 1:50,000 scale, HP = Helicopter Oblique Photography.

^c^Documentation status: PR = Previously Reported, UC = Uncertain.

^d^Non-systematic inspection.

^e^Includes 18.1 km^2^ of overlapping imagery.

a) the Central Culmí Valley, which corresponds to the main area of the Begley pedestrian survey and the headwaters of the Wampu River;
b) a small section of the North Culmí Valley that is north of the pedestrian survey area, along the headwaters of the Paulaya River, and where archaeological sites were easily identified on Google Earth imagery;
c) the Southern Culmí Valley, south of the pedestrian survey and along the course of the Wampu River Valley to the village of San José del Guano;
d) the Central Wampu, which is the main portion of the watershed east of San José del Guano to the point where the Wampu and Pao Rivers join, and which also includes part of the main Wampu tributaries (Aner, Ahuas, Lagarto, Guano);
e) the T4 lidar area, which consists of two small strips intersecting the Pao River close to the borders of Colón, Olancho, and Gracias a Dios; and
f) the T1 and T3 lidar areas in Gracias a Dios.

Hundreds of low confidence features were digitized in many of these areas, but because of the difficulty of validating these as potential sites without further data (remote or in situ), for this paper, we have chosen to only report and include in the analyses the medium and high confidence sites.

A total of 264 km^2^ of the western Wampu (where it runs predominantly north to south) was inspected on Google Earth pan-sharpened real color imagery with a resolution estimated at 50 cm. This area includes the main pedestrian survey area of roughly 92–105 km^2^ in the central Culmí Valley. Within this area, Begley reported 86 monumental sites, providing detailed site layout diagrams without geolocation information. One of the reported sites, PRP-4 or Marañones, has two distinct groups at different terraces levels (upper and lower) and with different architectural styles; thus, for our analysis, we considered these as two different sites, bringing the total Begley survey site count to 87. Our visual analysis of the area resulted in the identification of 7 sites, 4 at high confidence and 3 at medium; a detection rate of only 8.1% (Fig 4, Table 3). Importantly, although we are aware of the location of PRP-4 from topographic maps produced in the late 1960s and early 1970s, we were unable to identify it in several of the historical Google Earth images, highlighting the limitations of satellite imagery datasets (as illustrated in Fig 3). For all sites identified in the satellite imagery, only a small portion of the monumental structures are visible; because of this, only one of the sites could be matched to Begley’s site diagrams (see S1 File and Google Earth to interactively assess the limitations of VHR satellite imagery for the remote identification and mapping of archaeological features in eastern Honduras).

In the north Culmí Valley along the headwaters of the Paulaya River, we identified 5 sites (1 at high and 4 at low confidence) while performing the block assessment of the Google Earth imagery. These identifications were the result of chance encounters in the close vicinity of the grid-based assessment of the central Culmí region. Just to the south of the Begley survey block, near the intersection of the Wampu and Culuco Rivers, 4 sites were identified – one of considerable extent for the region (about 30 hectares), with multiple dispersed mounds in a prime agricultural area (see S2 File).

On the eastern extreme of the Wampu River system, we identified archaeological sites using airborne lidar data in two small valleys (T1 and T3) in Gracias a Dios, and one single mound site in the department of Colón (T4) based on an opportunistic lidar data collection (i.e., a single strip). In T1, 5 sites were previously reported by [15], but we classify site GD-955 as three different sites (GD248R969J, GD249R971J, GD244R962J) rather than one due to the open space and steep slopes between the architecture, for a reported total of 7 sites. For T3, we identified a total of 6 previously unreported complex sites at high and medium confidence.

One of the main contributions of this study is to virtually survey the gap along the Wampu River between Begley’s pedestrian survey and the lidar surveys of 2012, accomplished by visually inspecting the 50 cm resolution panchromatic band of the Worldview 2 & 3 imagery. This inspection led to the identification of 42 archaeological sites, 14 with high confidence levels and 28 at medium confidence. Of these sites, 3 have been previously reported, including Las Crucitas de Aner I, a large site identified on topographic maps and uniquely located away from the banks of any main river or stream [43], and a smaller site along the Ahuas River, known to IHAH as Mahor. Notably, we were not able to locate the known sites of Las Crucitas de Aner II, Porton del Cielo, Maikawana, and a site with two parallel mounds reminiscent of Caribbean and Mesoamerican-style ball courts that was recently identified by IHAH on a field visit.

### Patterns in site layouts

**Fig 5:**
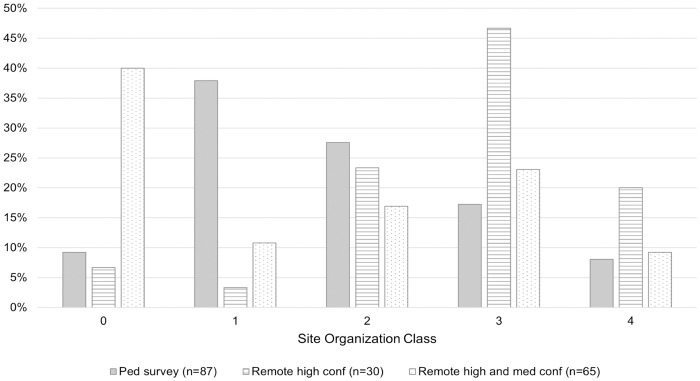
Classifications of site organization (0-4) by dataset type.

Using the site classes, 0–4, we present the results by dataset and confidence levels (Figs 2 and 5). To contextualize the architecture for the Wampu River system, we include all of Begley’s 87 sites (with PRP-4 considered as two different sites) in the study region, and 65 sites (72 minus 7 identified within the Begley pedestrian survey region) remotely-identified here at medium and high confidence levels via lidar and satellite data, for a total of 152 sites. We were also interested in looking at site organization by dataset in order to detect any patterns, biases, or limitations based on the type of data used. The sample of sites identified via pedestrian survey (n = 87) constitutes a control sample given its wall-to-wall continuity and high detection rate. In this sample, the most common classification was 1 (>1 mound with no grid-like organization), constituting 38% of the sample, followed by class 2 (<50% organization) at 28%, class 3 (>50% organization) at 17%, and with smaller numbers of class 0 (single mound) at 8% and class 4 (full grid organization) at 7%. For the sample of sites identified via satellite and lidar remote datasets (n = 65), the organizational classes differ based on whether the sample was limited to sites identified with high confidence (n = 30), or whether high and medium confidence sites were included (Fig 5). For the high confidence sites, the most common class (47%) was 3, often with architecture showing right angles and multiple connecting features. Approximately 23% and 20% of these sites could be categorized as 2 and 4 respectively, while smaller percentages (3% and 7%) showed no grid-like arrangements and/or a single mound. When the high and medium confidence sites were considered, many more sites were placed in the single mound category of 0 (40%), with the fewest sites in class 4, the most organized classification (9%).

By breaking down the site organization by dataset, we highlight biases in the data and the value of using multiple types of datasets for a region. For example, the large number of sites with no grid-like organization (class 1) in the pedestrian survey reflects the surveyor’s ability to evaluate potential anthropogenic features on the landscape that may not be clearly identifiable in remote sensing data. In contrast, when examining sites remotely, there are potentially high numbers of anthropogenic single mounds, but these were often assigned a lower confidence level since they could not be field verified (class 0). If we were able to field verify all of the individual mounds, the proportion of class 0 sites in the medium or even high confidence sample would increase. This reinforces the need to field verify even the highest resolution remote sensing data in archaeology.

When comparing only the remote sensing data, the medium and high confidence sites show a bias towards more organized sites (classes 2, 3, and 4). This indicates that site identification in remote datasets is biased towards those with clear anthropogenic features on a grid system. The higher numbers of single mounds (class 0) in the entire remote sensing dataset versus in the high confidence sample suggests that there are many more archaeological features on the landscape that must be field verified. While in some ways this is a limitation of using remote sensing data to identify sites, it also shows the potential of the approach for evaluating the extent of anthropogenic landscapes. In the case of the Wampu River system, this means that there are potentially many more archaeological sites on the landscape than the 72 that we identify, and these could be documented with both higher resolution remote sensing data (i.e., airborne lidar) and field verification.

In addition to classification of the sites based on their level of organization, we start to note some basic architectural patterns that repeat through the Wampu area, and arguably throughout the broader eastern Honduras cultural region. In a previous publication based on lidar data for the T1 area of La Mosquitia, we noted two types of arrangements [15]. The first was the arrangement of the earthen mounds in a rigid square pattern (this was referred to as Type A). The core of site GD-955 (identified here as GD248R969J (Fig 2n)) was the best example of this layout type for that lidar dataset. This almost perfect square layout is also observed at the previously reported site of Las Crucitas de Aner I (Fig 2m), and with less rigidity at site PRP-59 in the pedestrian survey and at the remotely identified site of Mahor (OL085R123I). The second arrangement that was identified was rectangular, defined by three mounds, with one mound forming the long dimension of the rectangle and two mounds on either of the shorter end of the rectangles. These arrangements were observed on satellite groups of the previously reported site GD-955, but here they are identified as individual sites GD249R971J and GD244R962J.

By expanding our sample with additional lidar data (T3), satellite imagery, and Begley’s site diagrams from the central Culmí valley, an emerging pattern is that the two square and rectangular-shaped arrangements are basic architectural blueprints of intentionally designed and built sites. Some sites with a few mounds may be arranged with a one of these patterns; for instance, site OL013R259I (Fig 2g) has only two identifiable mounds in the available satellite image, and they are arranged in a wide rectangular pattern, with the two mounds defining the short side of the rectangle. On the other extreme, complex sites such as OL135R108I (Fig 2o), the extended GD-955 from the T1 lidar collection, and PRP-11 (Suyapita) and PRP-39 from the pedestrian survey, have elements of both square and rectangular arrangements, in addition to parallelism (further expanded below).

Another common arrangement style, which we refer to as a “U” arrangement, is one in which three or more mounds are arranged in a U-shape. This arrangement is visible at site GD454R801J (Fig 2k) with a double U in inverted fashion, Las Crucitas de Aner “C” or OL101R173I (Fig 2l) with the south side defined by a long-curved mound, and perhaps most notably in the “upper group” of the previously reported site of Marañones (PRP-4) in the central Culmí Valley. Other sites in Begley’s pedestrian survey that exhibit this arrangement are PRP-71 and PRP-100. Also, while outside of the Wampu river system, the site of Culuco (Fig 3) exhibits a complex U arrangement.

**Fig 6:**
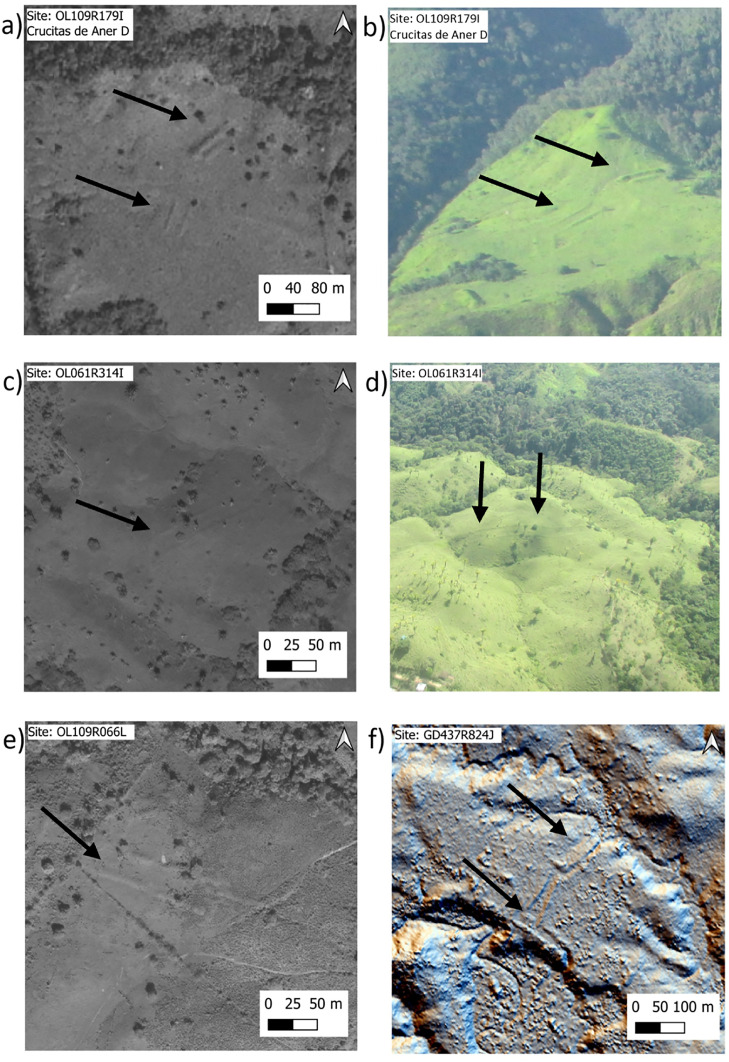
Sites with parallel architectural features, as discussed in the text. Images a, c, and e are from WorldView 2 and 3 satellite multispectral data (imagery used with permission from Maxar Technologies ©2025); b and d are helicopter photos by the authors; f is from airborne lidar scanning (image produced by authors from lidar data courtesy of UTL productions).

A final observation on architectural organizational styles through the broad region is parallelism. In his survey, Begley [14] noted sites with long parallel mounds with a narrow separation between them that were reminiscent of Mesoamerican ball courts. Similar elements are present in the core of site GD-955, as observed from the lidar visualizations originally published in [15]. However, as we increase our database of archaeological sites and their layouts in eastern Honduras, it is clear that there is variation in the complexity and diversity of parallel architectural features. This complexity and diversity are illustrated Fig 6, which shows imagery for four different sites with long narrow structures. Fig 6a and 6b are images from site OL109R179I, or Las Crucitas de Aner “D”. In the satellite image (Fig 6a), it is possible to identify two sets of parallel structures, two large but asymmetrical mounds with a narrow space in between to the north, and another set of parallel mounds towards the center of the image. In the oblique photo from the helicopter (Fig 6b), there are at least three sets of parallel structures with different azimuths that seem to contour to the topography. Figs 6c and 6d are of site OL061R314I, close to the Lagarto River, which includes a set of long and narrow parallel mounds along the top of a small hill with no other visible architecture. The architecture of this site/structure is reminiscent of Mesoamerican causeways delineated by similar narrow long mounds [65], but at this point, we cannot attribute function based simply on morphological similarities. The satellite image in Fig 6e is of site OL109R066L, which consists of three long narrow mounds, two of which are quasi-parallel and curving toward the outside. Finally, Fig 6f is a lidar-derived image of a site (GD437R824J) made out of three sets of long and narrow parallel mounds in a complex geometric arrangement. Within Begley’s pedestrian survey dataset, site PRP-3 has structures that form similarly long and narrow complex parallel arrangements. An interesting future step would be to document the variation in style and topographic locations of these parallel arrangements, in addition to any discussion about function (i.e., ballcourts).

### Site locations

**Fig 7:**
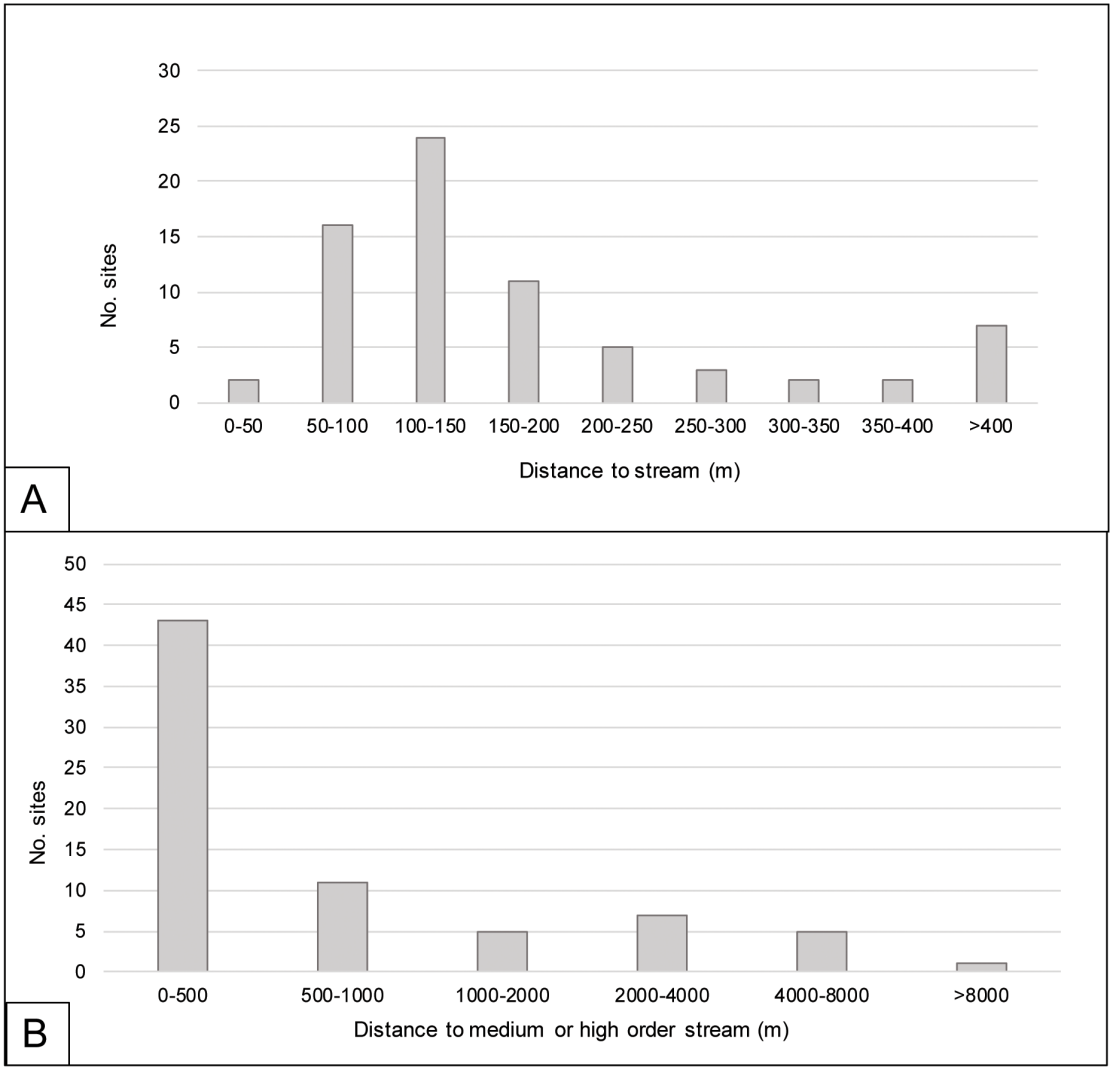
Site distance from streams. A. Histogram showing the distances (m) between the centroids of all 72 archaeological sites remotely identified and the closest stream centerline of any order. B. Histograms showing the distances (m) between the centroids of all 72 archaeological sites remotely identified and the streams of medium or high order only.

The results from the distance assessments for the 72 sites identified in the remote sensing data are presented in Figs 7A and 7B. The greatest number of sites (n = 24) are located 100–150 m away from any stream. Further, 80% of sites are located less than 250 m from a stream of any order, and 90% are less than 400 m away (Fig 7A). This does support the anecdotical observations and assumptions that sites are located near active water bodies. Importantly, however, many of these streams are of low order and probably only carry water during the rainy season. When we consider site distance to medium or higher order rivers only, some sites are located at considerable distances from the main water-carrying streams (Fig 7B). The vast majority (80%) of sites are located within 2 km from a main river, but 8% of sites are located at distances larger than 4 km. We identified 6 sites (8.3% of all sites remotely identified) that appear to be at the origin of the watershed, at distances that range from 200 to 2,300 m from a medium or high order stream. As we discuss below, the location of these sites at the watershed origin suggests that there are factors other than the accessibility of permanent running water that determine site location.

### Site orientation

**Fig 8:**
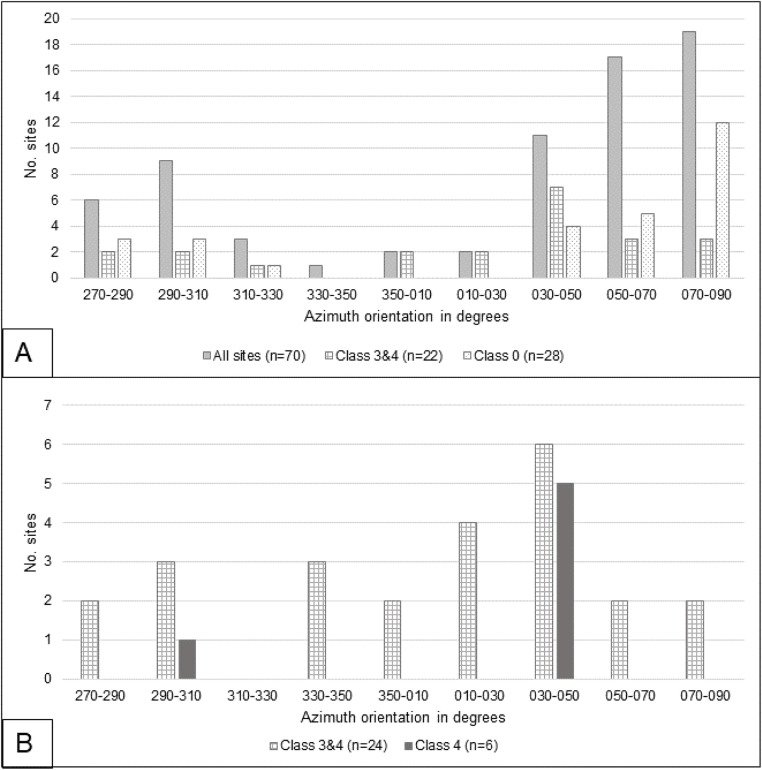
Site orientation in degrees. A. Histogram of azimuth orientation of the longest axis of the largest structure of the site for all remotely identified site. Two sites in classes 3 and 4 were not included because it was difficult to accurately determine the largest structure. B. Histogram of the azimuth of the site’s main axis for remotely identified sites of organization classes 3 and 4.

The main results from the orientation analysis are presented as histograms in Figs 8A and 8B. Fig 8A shows the histogram for the orientation of the main structure at twenty-degree intervals, segregated into three cases: a) all sites, b) sites that have an organization class of 3 and 4, and c) class 0 or single mound sites. A few interesting patterns are evident in this graph. First, there seems to be a preference for azimuths from 45° to 90°, with very few structures that are oriented North to South. Also, single mound sites seem to have a high preference for a West to East orientation (Fig 2a, 2b, and 2c). To further explore the orientation data, we plotted a histogram for the main site axis azimuth, also at twenty-degree intervals, and segregated them into two cases: a) sites that are class 3 and 4, and, b) only the sites with class 4 organization (Fig 8B). It is important to note that Fig 8B is generated with a small sample size (n = 24 for class 3 and 4 sites, n = 6 for class 4 sites). With this caveat, the histogram shows a mostly uniform distribution, with some preference for the northeast direction among the most organized sites. In contrast, for those sites with the clearest organization (class 4), the majority (5 out of 6) of the sites have a preferential orientation close to the 45° azimuth. Interestingly, a reinspection of the imagery showed that many of the sites with a square layout were constructed with a 45° azimuth orientation (Fig 2m and Fig 2n), while most sites with a rectangular layout (Fig 2j) and those with only a single mound favor an East to West orientation.

## Discussion

Since the sixteenth century, eastern Honduras has been noted in the archaeological and ethnohistoric literature as containing numerous undocumented archaeological sites [7,14,19,24,31,40,43,44,66,67]. Cultural geographer William Davidson [19] argued that the Pech, one Indigenous group in eastern Honduras, owed their survival of Spanish incursions to the location of their territories and settlements on the “edge of colonial success,” surrounded by rugged topography and streams inaccessible to nonnative watercraft. Within the context of the Wampu River system data presented here, topography and water are particularly interesting points for a discussion about site and architectural chronology, settlement patterns, and the types of anthropogenic landscapes that characterized precontact eastern Honduras.

### Chronology and settlement patterns

Scholars note that eastern Honduras should be understood on its own, comprised of Indigenous landscapes with diverse architecture and settlement patterns [7,10,68]. Based on the number and types of site organization documented here, there are diverse kinds of features and architecture. One possibility is that some of this diversity may have more to do with chronology than with intra-regional variation. IHAH links monumental architecture with Cocal period (AD 1000–1521) occupations [43,44]. Thus, some of the sites with long parallel features that look like Caribbean and Mesoamerican-style ballcourts and large open spaces (i.e., plazas) flanked by earthen mounds may be associated with the late phase of precontact populations. Following this logic, sites with more formal architectural arrangements, such as organizational classes 3 and 4 presented here, may be linked to the Cocal period as well. Previous studies link the earlier Selin period to sites with fewer formal arrangements, such as variously sized mounds with no apparent grid-like arrangement (e.g., Talgua Village; Selin Farm). Based on observations by Strong [31], Cuddy [7] suggests that these earlier sites represent a regional phenomenon of nucleated mounded architecture in a defensive position. Following this interpretation, the subsequent Transitional Selin to Early Cocal populations began to settle in a more dispersed manner, and this may reflect broader sociopolitical transitions throughout Central America. At this point, we cannot say whether the organizational diversity we see is a reflection of a nucleated to dispersed settlement pattern.

Another possible avenue for exploring site organization and chronology could be cardinal alignments. In the data presented here, we observed that a significant portion of the largest structures have East-West alignments and that sites with square versus rectangular rigid alignments prefer certain orientations. Using a much larger dataset in the Mesoamerican Gulf Coast, Šprajc et al. ([60]; see also [61]) applied airborne lidar data to document patterns in architectural orientation dating to the Formative and Late Classic periods (c. 1050 BC – AD 1000). Numerous buildings were oriented to both the sun’s position on significant dates and a structure in the same direction, suggesting astronomical intentionality. They argue that these orientations reflect differing cultural traditions over time that increasingly facilitated the use of observational calendars, enabling the Maya and their ancestors to schedule seasonal activities and rituals. In eastern Honduras, the square and rectangular alignments may represent different cultural traditions, as argued for Mesoamerica, or simply different settlement patterns over time. With a larger sample, future study on architectural orientations in eastern Honduras could provide an interesting comparative sample to the Mesoamerica datasets.

Beyond chronology, the data on water distance suggest that physical geography played a role in settlement location. Certainly, site location near water is expected, but the sites that are further away from water, or are close only to low order, seasonal water bodies, are interesting. The association between seasonality and settlement location at both coastal and inland locations was observed in northeastern Pech settlements in the 1940s by Doris Stone [30], but also more recently by Davidson [19]. Their conversations with Indigenous communities indicate that people in the region moved based on water patterns, including whether constructed canals were usable between coasts or rivers and sites. Today, parts of Honduras receive 1,500 mm of rain annually, but there is a marked dry season between January and May. European observations in the sixteenth century also noted that many settlements were seasonal, with inland communities in particular moving during high and low water flow periods [24]. Future work that explores whether the hydrographic network has been stable or changing over the past 500 years would further evaluate the potential relationship between site locations and seasonality.

There are other possible reasons why precontact populations constructed sites where they did in relation to water bodies. For example, boundary maintenance vis-à-vis water bodies may have been important for regional settlement patterns. Davidson [19] and colleagues tested this idea by venturing down the Wampu River near the modern boundary of the Pech and Sumu (Tawakha) territories, and they found that they could not pass the largest of the rapids safely, and that a “canoe line” separates the upstream Pech from the downstream Tawakha. Modern Pech people live in the highest watersheds, and if this was the case around Spanish arrival, this challenging topography may have been a reason why some communities evaded Spanish incursions [19]. On a much smaller scale, these observations about site location away from certain water bodies is comparable to findings in the Amazon, where mounting evidence records the presence of anthropogenic soils and some archaeological sites in interfluvial areas away from major rivers [12,69–71]. Similar to the Amazon datasets, precontact communities in the Wampu River region likely settled the landscape, especially in relation to water bodies, for a variety of environmental and cultural reasons.

Finally, the location and ability to cultivate agricultural plots probably played a major role in anthropogenic landscapes and settlements. Some sites in our sample, such as those located at the watershed origin and away from flowing water, could also be placed based on where the soil best absorbs rainfall, making the land fertile for agricultural or even construction purposes. A discussion of agriculture is beyond the scope of the datasets presented here, but there are hints for future research. Ethnohistoric observations indicate that the Pech moved seasonally for water, but also because of changing soil fertility and the usability of agricultural plots [24]. In the Culmí Valley and along the Talgua River, settlement locations are on higher terraces where the terrace was narrow, but close to an area where it widened. It is possible that the wider terraces were used for agricultural activities ([14]; see also [73]), such as at GD-955 in the Mosquitia [15]. Elsewhere in Honduras, during the dry season, irrigation is necessary, and there are visible canals from these kinds of terraces in the remote sensing data, as well as at the Early Cocal Río Claro site in northern Honduras [34,35] (Fig 1). These hints at wider-scale agriculture suggest that it should be yet another factor for identifying and interpreting the locations of precontact sites in eastern Honduras.

### Tropical settlement patterns and urbanism

In recent years, archaeologists have increasingly made the case that in tropical regions, settlements were dispersed along diverse topographies and river systems, and that many of these settlements were agrarian-based and connected informally to a larger ritual center [74–77]. As Hawken and Fletcher [75] point out, these low-density dispersed urban settlements were a reoccurring feature of past societies, especially in tropical landscapes. Low-density urbanism has been more broadly discussed in recent years due to changing notions of urbanism, but also because of wide-area lidar data in southeast Asia [78–80], the Amazon [5,81] (see also [12,81]), and several parts of the Maya region in Mexico and Central America [11,83,84]. These studies and others address long-held assumptions about human-environment interactions in tropical locations, many of which have impacted archaeological work and interpretations. In tropical locales, Elizabeth Graham [85] noted that colonialists had difficulty with the climate, and the poor preservation of material culture was interpreted as an absence of phenomena. There was also a prejudice against trees as a barrier to more extensive survey coverage. The combination of lidar data, changing notions of urbanism, and some integration of traditional ecological knowledge has changed our assumptions about the role of greenspace and water in tropical archaeological landscapes.

Although this study is preliminary, the eastern Honduras archaeological data have the potential to contribute to discussions of tropical settlement patterns, and possibly tropical urbanism. The region is a tropical ecozone, and archaeological features are dispersed throughout river systems and beyond, with populations probably relying on varied adaptive strategies. The diverse architectural organization, site locations near water systems, and orientation patterns all point to similarities with other tropical settings in which people transformed landscapes through small-scale hydraulic and agricultural management, with open spaces and gardens [72,74,77,82,86]. In eastern Honduras and elsewhere, communities of farmers would have relied on various subsistence strategies, which involved small-scale production and exchange, and water and agricultural systems. We cannot discuss scale or population sizes without higher resolution geospatial data, and data obtained from traditional field archaeology, but we note that tropical settlement patterns, including those referred to as urban, vary considerably in size and in architectural type. For example, wide-area lidar data in the Ecuadorian Amazon demonstrates that the region was characterized by a type of garden urbanism, in which more than 6,000 dispersed earthen mounds were connected by road networks and gardens over 2,000 years [5]. This is in contrast to enormous populations (e.g., 750,000 people) living over 1,000 km^2^ in stone and earthen architecture in the Greater Angkor region of Cambodia [79].

The Wampu River system data highlight the potential for reevaluating greenspace for anthropogenic features in tropical Honduras. We maintain that discussions about tropical settlement patterns should include lesser-studied regions like eastern Honduras, because omitting this area and others would do a disserve to the broader understanding of complexity and urbanism in tropical ecozones. It is important that we investigate the wide array of tropical urban experiences if we want to address how people created communities and modified landscapes in the past, and the types of organization and innovation that persisted or failed.

## Conclusions

The main goals of this study are to integrate remote and pedestrian survey datasets and to identify any patterns in the location and layout of sites within the Wampu River system. By combining these datasets, we make three contributions to the remote sensing and archaeological literature.

First, we present the first broad survey and synthesis of >650 km^2^ of archaeological data in eastern Honduras, connecting two previously reported surveys at opposite ends of the Wampu River system. Archaeological research in this part of the Americas has been inconsistent, so bringing together different datasets is a contribution in itself. Methodologically, the practice of aggregating data collected from historic maps, pedestrian survey, airborne lidar survey, satellite images, and oblique helicopter photos demonstrates the potential of multiple and diverse datasets to document and to understand, at a broader scale, the archaeology of a region. This is especially crucial in threatened regions, such as eastern Honduras, but it is important globally as well. We also show the limitations of the different datasets, concluding that future work should integrate both high resolution lidar data and field verification when possible.

Second, we identify archaeological sites within the context of previous work, legacy datasets, and new data collections. Within the Wampu River system, we identify 72 sites, 55 of which were previously unknown in the archaeological literature. This represents a contribution to the documentation of heritage in a rapidly changing part of Honduras. After these initial steps of identifying and quantifying archaeological sites, scholars can work towards analyzing sites over a region to understand change spatially and over time, as has been done in more research-rich regions like the Maya cultural area.

Finally, this study has identified and provided basic analysis of architectural and site patterns over the Wampu River system. Using a basic classificatory scheme, we identified patterns in site configurations based on whether features were organized on a grid system. Nearly half of the sites that were identified as high or medium confidence sites could be classified as highly (>50%, within the 3 and 4 classes) organized sites, often with multiple structures on a grid system. We highlighted three architectural configurations that appear throughout eastern Honduras – square, rectangular, and parallel – and which can contribute to future architectural typologies in the area. This approach also shows the limitations in the types of datasets used, with a bias towards less organized single or double mound sites in the pedestrian survey, and with more highly organized sites most visible via remote sensing data.

The data showing site distances from water sources indicate that, while most sites are near continuous water sources, some are either near seasonal water supplies or located at the source of watersheds. These results highlight the possibility that settlements were seasonal and/or that site locations were selected for reasons other than their proximity to water sources. Locations could have been selected for strategic trading and political purposes, or for proximity to agricultural plots and fertile soils. In addition, preliminary site orientation data show a preference for east-west orientations, with some initial observations regarding architecture with square versus rectangular configurations. Previous work in other regions has shown that humans have long organized their settlements according to seasonal cycles and astronomical patterns [86,87]. Similar research in eastern Honduras could provide broader regional comparisons regarding differing cultural traditions and chronology.

The diversity of settlements, the use of rivers and stream confluences, and the hints of agricultural activities within the region all serve as potential points for future research. Throughout Central America and in non-Maya Honduras specifically, there has been a long-standing observation that internal diversity, rather than outside cultural influences, characterized polities and the archaeology of the area [7,50,69,88]. We hope that this study can serve as a model for future work on landscape modification, earthen mounds, chronology, and tropical settlement patterns. We acknowledge that significant energy is necessary to bring together such diverse datasets for archaeological purposes—but, in our era of threatened Indigenous landscapes, the task has never been more critical.

## Supporting information

S1 File Google Earth.kmz file from Culuco, Olancho. Researchers can use this file to compare and contrast a UAV-derived lidar shaded relief map of an archaeological site with the historical VHR satellite imagery accessible via Google Earth. The layers in the.kmz file include: a) an outline showing the surface area depicted in Fig 3; b) outlines of four archaeological structures as interpreted by the authors by analyzing VHR Google Earth imagery from March 2018, April 2019, September 2020, and April 2025; c) bare-earth shaded relief map generated from UAV lidar data of the site.
(KMZ)

S2 File Google Earth.kmz file which includes illustrative data of the methodology and results from the present study made available for reproducibility purposes. The.kmz file includes the following layers: a) outlines of the areas for which purchased WorldView 2 and 3 images were analyzed for the middle Wampu river section; b) outline for the area analyzed in the Culmí Valley and western Wampu river section; c) 500 m x 500 m inspection grid for the Culmí Valley and western Wampu river section; d) approximate location for the pedestrian survey blocks carried out by Begley in the late 1990s; e) centroids and structure outlines for all the high and medium confidence site identified in the Google Earth VHR imagery within the Culmí Valley and western Wampu river section; f) samples of a few features (outlines) that could be archaeological in nature, but for which the authors identified at low confidence and thus not accounted for or reported in the present publication.
(KMZ)
